# Meta-analysis of budesonide and surfactant combination for the prevention of bronchopulmonary dysplasia in preterm neonates based on gestational age

**DOI:** 10.3389/fped.2025.1518957

**Published:** 2025-04-24

**Authors:** Sedigheh Ekraminasab, Mahmood Noorishadkam, Hossein Neamatzadeh, Mohamad Hosein Lookzadeh, Seyed Reza Mirjalili, Mahta Mazaheri, Seyedeh Elham Shams

**Affiliations:** ^1^ Mother and Newborn Health Research Center, Shahid Sadoughi University of Medical Sciences, Yazd, Iran; ^2^ Department of Hematology and Blood Banking, School of Allied Medical Sciences, Shahid Beheshti University of Medical Sciences, Tehran, Iran; ^3^ Department of Pediatrics, Hamadan University of Medical Sciences, Hamadan, Iran

**Keywords:** bronchopulmonary dysplasia, pulmonary surfactant, budesonide, controlled trials, preterm neonates

## Abstract

**Background:**

Budesonide, an inhaled corticosteroid, and surfactant, a substance that lowers surface tension in the lungs, are both used to prevent Bronchopulmonary Dysplasia (BPD). This meta-analysis evaluates the effectiveness of combining budesonide and surfactant in preventing BPD in preterm neonates compared to surfactant alone.

**Method:**

A comprehensive search of electronic databases, including PubMed, Scopus, Google Scholar, CNKI, and Embase, was conducted from their inception up to August 30, 2024. The focus was on evaluating the combination of Budesonide and surfactant for the prevention of BPD in preterm neonates. This assessment involved calculating ORs and their 95% CIs to determine the treatment's effectiveness. The primary outcomes measured were the incidence of BPD and mortality rates, while secondary outcomes included the rates of intraventricular hemorrhage (IVH), retinopathy of prematurity (ROP), patent ductus arteriosus (PDA), sepsis, neonatal necrotizing enterocolitis (NEC), and pneumothorax.

**Results:**

This research, combining a meta-analysis and observational data, indicates that Budesonide-Surfactant therapy significantly reduces BPD in preterm neonates with NRDS, regardless of gestational age. Additional benefits, including decreased mortality (in ≥27 gestational weeks), NEC, PDA, ROP, and Sepsis, were observed in the observational study, though pneumothorax increased in the ≥27 gestational weeks group. The meta-analysis corroborated reductions in BPD, PDA, and mortality (in ≥27 gestational weeks), supporting the potential of Budesonide-Surfactant to improve outcomes in preterm infants.

**Conclusions:**

The intratracheal administration of pulmonary surfactants combined with budesonide was associated with a reduction in the incidence of BPD, mortality, and PDA. Although the prevalence of ROP, NEC, IVH, and sepsis was lower in the test group compared to the control group, these differences did not reach statistical significance. These findings suggest that the combined use of budesonide and surfactant is effective in preventing BPD and mortality, as well as in reducing certain secondary outcomes.

## Introduction

Bronchopulmonary dysplasia (BPD) is a chronic lung disease affecting primarily preterm infants, characterized by impaired lung development and long-term respiratory issues due to factors such as mechanical ventilation, oxygen toxicity, and inflammation (1). The understanding of BPD has evolved to differentiate “Old BPD,” marked by significant airway epithelial damage and fibrosis from oxygen toxicity and mechanical ventilation, from “New BPD,” which emphasizes impaired alveolar development and abnormal pulmonary vascularization that lead to ineffective gas exchange (2). Contributing risk factors include hyperoxia, causing cellular damage through reactive oxygen species, trauma from mechanical ventilation, and systemic inflammation that disrupts normal lung repair (3). Annually, about 10,000 neonates in the U.S. are affected, with up to 80% of those born at extremely low gestational ages developing BPD (4, 5). Global rates vary from 10% to 89%, depending on regional healthcare practices (6). Key risk factors include lower gestational age, low birth weight, lengthy invasive mechanical ventilation, oxygen exposure, maternal conditions such as chorioamnionitis and smoking, and socioeconomic disparities that lead to uneven morbidity rates among infants (7).

To prevent BPD, several effective strategies have been identified. Non-invasive ventilation methods, especially continuous positive airway pressure (CPAP), are preferred to mitigate lung injury associated with invasive mechanical ventilation, with studies showing that early CPAP implementation can reduce BPD rates (8). Surfactant therapy, particularly via the INSURE technique, decreases the need for mechanical ventilation and lowers BPD risk. The early use of inhaled corticosteroids like budesonide can help reduce inflammation and improve lung outcomes (9). Caffeine administration is also supported as a preventive measure for infants born at or before 30 weeks gestation. Antenatal corticosteroids are recommended for at-risk mothers, enhancing survival rates for preterm infants, even if they do not directly lower BPD rates (10). Maintaining optimal oxygen saturation levels between 90% and 95% is vital to avoid hyperoxia, which can increase BPD risk, and adopting volume-targeted ventilation strategies can further reduce ventilator-induced lung injury (11).

Recent studies have generated interest in budesonide-surfactant combination therapy as a preventive measure against BPD in neonates, targeting both inflammation and impaired lung development (12). Evidence suggests that the anti-inflammatory properties of budesonide, paired with surfactant therapy, may reduce BPD incidence and severity, leading to improved respiratory outcomes. Early administration of this combination has shown links to better lung function and reduced mechanical ventilation needs, although the findings are not universally consistent (13). A meta-analysis emphasizes the need for rigorous clinical trials across diverse populations to provide evidence-based recommendations for this treatment. Their review indicated that intra-tracheal administration of this combination could decrease both BPD incidence and the combined outcome of death or BPD in very low birth weight (VLBW) infants, although larger trials are necessary before standard care recommendations can be made (14). Additionally, safety evaluations suggest that intratracheal surfactant with budesonide does not elevate short-term complication risks while potentially reducing BPD incidence and mortality (15). Other preventive strategies, such as early surfactant therapy through the INSURE method, are also shown to minimize ventilator-associated lung injury (16). Evidence indicates that inhaled corticosteroids like budesonide, especially combined with surfactant therapy, may improve lung function and reduce inflammation without systemic side effects (17).

Integrating budesonide with surfactant therapy offers a promising avenue for reducing BPD in preterm neonates. While evidence supports its effectiveness, more research is needed to determine optimal dosing, timing, and long-term outcomes. A comprehensive approach, including non-invasive ventilation and close monitoring, is critical for enhancing results in this vulnerable population. Despite the proven benefits of budesonide-surfactant therapy, its routine use in neonatal intensive care units (NICUs) remains limited, underscoring the necessity for further investigation. This meta-analysis aims to update the efficacy of budesonide-surfactant therapy in preventing BPD and mortality in preterm neonates and examine its effects on secondary outcomes such as retinopathy of prematurity (ROP), patent ductus arteriosus (PDA), intraventricular hemorrhage (IVH), necrotizing enterocolitis (NEC), sepsis, and pneumothorax, while addressing barriers to its widespread implementation in NICUs globally.

## Materials and methods

### Search strategy

The research methodology involved a thorough and systematic examination of various online databases to gather relevant literature, utilizing major platforms such as PubMed, Web of Science, Europe PMC, ResearchGate, Elsevier, Cochrane Library, EMBASE, and SciELO. Additionally, databases dedicated to Chinese medical literature were included, such as Wanfang Data, Chaoxing, the Chinese Medical Citation Index (CMCI), VIP Information Consulting, Chinese Medical Current Contents (CMCC), the Chinese Biomedical Database (CBD), Sinomed, MEDREX, and the China/Asia On Demand (CAOD) service. The Weipu Periodical Database from the Chinese National Knowledge Infrastructure (CNKI) was also part of the review process. The search, which had a cutoff date of August 30, 2024, specifically focused on case-control studies investigating the effects of Budesonide and Surfactant Combination on the prevention of BPD. The search strategy involved a well-designed query with keywords related to BPD, including “surfactant,” “budesonide” (or “Budezonide”), “respiratory distress syndrome” (RDS), “Bronchopulmonary Dysplasia,” “Infant,” “Neonates,” “Preterm,” and “Chronic Lung Disease.” To ensure a comprehensive review, a manual search of references from pertinent articles and reviews was performed to identify additional studies not included in the database searches, encompassing both English and Persian publications. Notably, informed consent was not required for this meta-analysis, as the study solely analyzed existing literature, thereby adhering to ethical guidelines concerning participant consent.

### Inclusion and exclusion criteria

This meta-analysis included studies based on specific criteria: participants were required to be premature infants under 32 weeks of gestational age or classified as VLBW (less than 1,500 grams). The intervention involved the use of pulmonary surfactant combined with budesonide for treating NRDS, while the control group received pulmonary surfactant alone. Only original research articles, such as randomized clinical trials, cohort studies, and case-control studies, were considered for inclusion. Systematic reviews, narrative reviews, letters to editors, commentaries, preprints, abstracts, and findings that could not be retrieved in full text were excluded. Additional exclusions included case reports, case series, editorial correspondence, reviews, animal studies, *in vitro* experiments, conference abstracts, and other meta-analyses, as well as research deemed irrelevant to the topic. Studies with inadequate sample sizes for ensuring statistical validity or those reporting outcomes unrelated to BPD were also excluded.

### Data extraction

Two researchers conducted a systematic review by rigorously evaluating bibliographies of relevant studies using strict inclusion and exclusion criteria. They collected and cross-validated data for accuracy and reliability, resolving disagreements through discussion or by consulting a third scientist. The review began with an assessment of titles and abstracts to eliminate irrelevant studies, followed by a thorough examination of full texts to confirm eligibility. The extracted data included the first author, year of publication, study country, number of patients, gestational weeks, birth weight, and details of the intervention or treatment.

### Quality assessment

The quality of the included randomized controlled trials (RCTs) was evaluated utilizing the Cochrane Collaboration's risk of bias assessment tool. For observational studies, the Newcastle-Ottawa Scale was implemented to gauge their methodological quality. Based on these evaluations, the studies were classified into three categories: low, moderate, or high quality. This systematic assessment ensured a comprehensive understanding of the reliability of the findings and the potential implications for clinical practice and future research.

### Statistical analysis

The study investigated the use of a combination of Budesonide and surfactant to prevent BPD in preterm neonates by calculating odds ratios (ORs) and their 95% confidence intervals (CIs) to examine the effectiveness of the treatment. Statistical significance was assessed using a *Z*-test to compare the population mean with the sample mean, a method well-suited for meta-analysis that combines results from multiple studies for more robust conclusions. A Chi-square test evaluated heterogeneity, with significant heterogeneity indicated by *p* < 0.05. The *I*^2^ statistic was used to quantify heterogeneity, and a random-effects model (DerSimonian-Laird method) was applied when heterogeneity exceeded 50%, while a fixed-effect model (Mantel-Haenszel method) was used for low heterogeneity (*I*^2^ ≤ 50%). Distinguishing between fixed and random effects models is essential for accurate data interpretation, with fixed effects suitable for constant, time-invariant heterogeneity and random effects addressing variations across studies. Model selection must consider unobserved heterogeneity to avoid biasing random effects estimates if correlated with predictors, validated by tests like the Hausman test. Sensitivity analysis was conducted by excluding one study at a time to assess the robustness of findings and to identify studies that significantly affect effect size estimates, emphasizing the importance of transparency regarding shifts in effect sizes or directions. Publication bias was examined through Begg's and Egger's tests and visual funnel plot inspections for asymmetry; if bias was found, the trim-and-fill method was employed for adjustments. Data synthesis utilized Comprehensive Meta-Analysis (Version 4.0) software (Biostat, USA), with statistical significance set at a two-tailed *p*-value of less than 0.05.

## Results

### Characteristics of eligible studies

**Figure 1:**
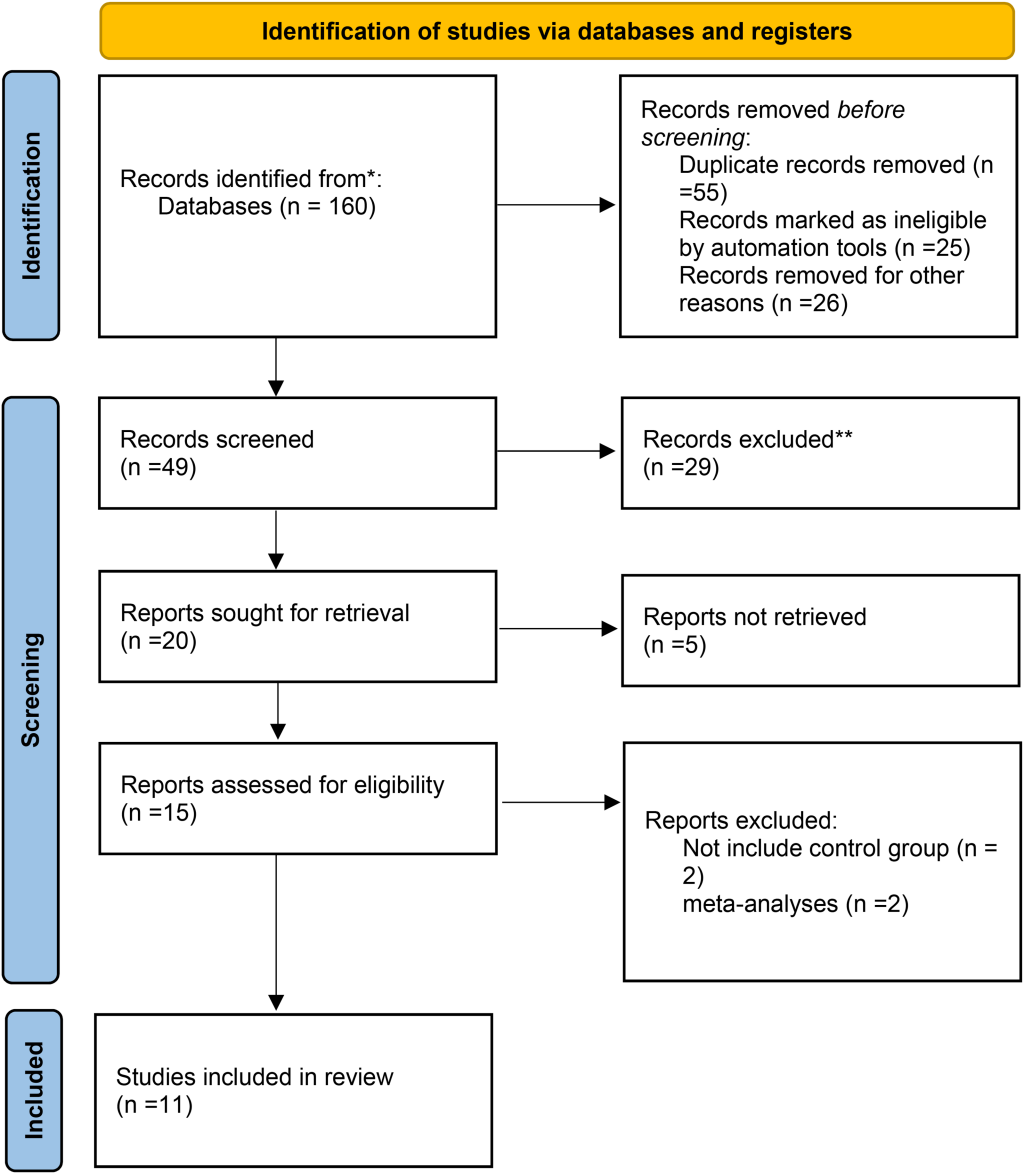
Flow chart of study selection on budesonide and surfactant combination for prevention of BPD in preterm infants.

An initial search identified 160 articles, leading to the review of 49 after duplicates were removed, resulting in the selection of 11 randomized clinical trials (12, 15, 18–23) and observational studies (24–26) for detailed examination (Figure 1). These studies primarily focused on VLBW or premature newborns, with an average gestational age of less than 29.3 weeks and mean birth weight under 1,562 grams. The main aim was to evaluate the effects of administering budesonide-surfactant on the incidence of BPD, mortality, and secondary outcomes compared to surfactant alone. A total of 2,137 subjects participated, including 945 in the test group and 1,192 in the control group. The eligible studies encompassed diverse interventions across various countries, including the USA, Taiwan, Iran, South Korea, and Italy, with a total of 2,137 patients. Most infants had gestational ages ranging from less than 28–37 weeks and birth weights predominantly below 1,500 grams. The primary intervention involved administering budesonide in conjunction with surfactants such as Beractant, Poractant alfa, Calfactant, and Curosurf, typically at a dosage of 0.25 mg/kg, while control groups received standard surfactant therapy without budesonide. The studies reflected considerable variation in treatment protocols and demographics, underlining the differences in clinical practices for managing respiratory distress in preterm infants (Table 1).

**Table 1:** Key features of randomized controlled trials and observational studies in the meta-analysis.

Author/year	Country	Sample size	Gestational age (weeks)	Birth weight (g)	Intervention/treatment	Test usage and dosage	Control usage and dosage
Yeh et al. (18)	Taiwan/USA	131/134	26.5/26.8	<1,500/<1,500	Budesonide 0.25 mg/kg + Beractant 100 mg/kg	Budesonide 0.25 mg/kg + Beractant 100 mg/kg	Beractant 100 mg/kg
Gharehbaghi et al. (12)	Iran	64/64	<30/<30	<1,500/<1,500	Poractant alfa 200 mg/kg + Budesonide 0.25 mg/kg	200 mg/kg Poractant alfa + 0.25 mg/kg budesonide	Poractant alfa 200 mg/kg
Heo and Jeon (19)	South Korea	16/18	28 ± 1/28 ± 2	<1,500/<1,500	Calfactant 105 mg/kg + Budesonide 0.25 mg/kg	Calfactant 105 mg/kg + 0.25 mg/kg budesonide	Calfactant 105 mg/kg
Moschino et al. (20)	Italy	18/18	<28/<28	<1,500/<1,500	Curosurf 200 mg/kg + Budesonide 0.25 mg/kg	Curosurf 200 mg/kg + 0.25 mg/kg budesonide	Curosurf 200 mg/kg
Kothe et al. (25)	USA	173/294	26.7 ± 2.1/26.7 ± 2.1	<1,250/<1,250	Budesonide 0.25 mg/kg + Beractant 100 mg/kg	Budesonide 0.25 mg/kg + Beractant 100 mg/kg	Beractant 100 mg/kg
Baghal Safa et al. (21)	Iran	35/35	29.34 ± 2.19/29.94 ± 2.11	1,139.86 ± 230.28/1,186 ± 224.14	2.5 cc/kg of Curosurf solution + 250 μ/kg of Palmicort	2.5 cc/kg Curosurf + 250 μ/kg Palmicort	2.5 cc/kg of Curosurf solution
Marzban et al. (22)	Iran	67/67	31.66 ± 2.84/31 ± 2.96	1,584.55 ± 505.02/1,465.45 ± 520.88	NA	NA	NA
Armanian et al. (15)	Iran	95/95	28.94 ± 1.57/29.015 ± 1.57	1,134.97 ± 237.61/1,190 ± 289.33	Each infant received Budesonide once at a dose of 0.25 mg/kg (0.5 cc/kg)	0.25 mg/kg budesonide	200 mg/kg
Anderson et al. (26)	USA	173/294	26.7 ± 2.1/26.7 ± 2.1	≤1,250/≤1,250	Budesonide 0.25 mg/kg mixed with Beractant surfactant 4 ml/kg	Budesonide 0.25 mg/kg + 4 ml/kg surfactant	4 ml/kg surfactant
Kashaki et al. (24)	Iran	148/148	<37/<37	<1,500/<1,500	Surfactant (Braksurf) + Budesonide 0.25 mg/kg	Surfactant + 0.25 mg/kg budesonide	4 cc/kg surfactant (Braksurf)
Habibi and Toohidinia (23)	Iran	25/25	32.4/30.4	3,326/1,504.2	Intratracheal Survanta (1 milliliter) + Budesonide (0.25 milligram)	Intratracheal Survanta + 0.25 mg budesonide	Intratracheal Survanta

In this table, the left numbers represent the test group (surfactant and Budesonide) and the right numbers represent the control group (solely of surfactant.).

### Outcomes of budesonide-surfactant therapy in neonates with NRDS stratified by gestational age

This study compared the efficacy of Budesonide-Surfactant vs. Surfactant alone in neonates with Neonatal Respiratory Distress Syndrome (NRDS) across two gestational age categories: ≥27 gestational weeks (Table 2) and ≤27 gestational weeks (Table 3). In the ≥27 gestational weeks group (*n* = 2,137), significant reductions were observed in the Budesonide-Surfactant group compared to the Surfactant alone group for several primary and secondary outcomes. Specifically, the proportion of BPD was significantly lower in the Budesonide-Surfactant group (31.0%) compared to the Surfactant alone group (54.3%) (*p* < 0.001). Additionally, the Budesonide-Surfactant group demonstrated lower proportions of mortality (8.2% vs. 11.2%, *p* = 0.012), NEC (5.6% vs. 9.1%, *p* = 0.029), PDA (21.4% vs. 22.9%, *p* = 0.009), ROP (3.8% vs. 6.9%, *p* = 0.011), and Sepsis (1.7% vs. 3.1%, *p* = 0.047). However, the proportion of Pneumothorax was higher in the Budesonide-Surfactant group (2.8% vs. 1.5%, *p* = 0.046). No significant difference was found in the proportion of Intraventricular Hemorrhage (IVH) ≥ between the two groups (*p* = 0.672).

**Table 2:** Comparison of primary and secondary outcomes in neonates with NRDS between budesonide-surfactant and surfactant alone (gestational age ≥27).

Study	Sample size	Mortality		BPD		IVH ≥		NEC		PDA		ROP		Sepsis		Pneumothorax	
		Test	Control	Test	Control	Test	Control	Test	Control	Test	Control	Test	Control	Test	Control	Test	Control
Gharehbaghi et al. (12)	128	6	9	24	38	2	4	13	17	2	3	21	25	1	3	0	0
Heo and Jeon (19)	34	1	4	5	8	1	1	0	0	1	3	2	5	0	0	0	0
Moschino et al. (20)	36	1	0	8	9	0	0	1	3	0	0	10	11	0	0	0	0
Baghal Safa et al. (21)	70	4	9	3	14	5	2	6	12	19	26	22	12	0	0	8	5
Marzban et al. (22)	134	10	19	8	10	9	16	0	0	0	0	7	6	0	0	0	0
Armanian et al. (15)	190	13	15	46	48	59	45	20	20	38	47	37	41	9	0	22	9
Kashaki et al. (24)	296	3	5	4	13	0	0	6	11	0	0	0	0	0	0	0	0
Habibi and Toohidinia (23)	50	3	5	0	0	0	0	0	0	0	0	0	0	0	0	0	0
Total	2,137	88	120	331	580	132	121	60	97	229	245	41	74	18	33	30	16

**Table 3:** Comparison of primary and secondary outcomes in neonates with NRDS between budesonide-surfactant and surfactant alone (gestational age ≤27).

Study	Sample Size	Mortality		BPD		IVH ≥		NEC		PDA		ROP		Sepsis		Pneumothorax	
		Test	Control	Test	Control	Test	Control	Test	Control	Test	Control	Test	Control	Test	Control	Test	Control
Yeh et al. (18)	265	17	22	38	67	0	0	0	0	0	0	0	0	0	0	0	0
Kothe et al. (25)	467	15	1	74	155	27	48	11	21	90	185	6	2	11	22	0	0
Anderson et al. (26)	467	15	31	121	218	27	48	0	0	0	0	26	52	0	0	0	0

Test (surfactant + Budesonide); Control (surfactant).

In the ≤27 gestational weeks group (*n* = 1,199), the Budesonide-Surfactant group also exhibited a significantly lower proportion of BPD (38.9%) compared to the Surfactant alone group (73.4%) (*p* < 0.001). Similar reductions were observed in PDA (15.0% vs. 30.9%, *p* < 0.001), IVH ≥ (9.0% vs. 16.0%, *p* = 0.002), NEC (1.8% vs. 3.5%, *p* = 0.027), ROP (5.3% vs. 9.0%, *p* = 0.011), and Sepsis (1.8% vs. 3.7%, *p* = 0.029). However, no significant difference in mortality was observed between the groups (*p* = 0.227). No cases of pneumothorax were reported in either group. These findings suggest that Budesonide-Surfactant may offer significant benefits in reducing BPD, NEC, PDA, ROP, and sepsis in neonates with NRDS, regardless of gestational age, although the incidence of pneumothorax was increased in the budesonide group for neonates ≥27gestational weeks.

### Quantitative data synthesis

#### Primary outcomes

##### BPD incidence

**Figure 2:**
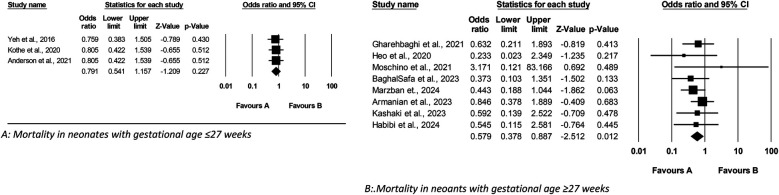
Meta analysis of the role of budesonide-surfactant mixture versus surfactant alone in **mortality** rate in preterm neonates with NRDS. **(A)** Mortality in neonates with gestational age ≤27 weeks. **(B)** Mortality in neoants with gestational age ≥27 weeks.

Figure 2 presents a meta-analysis examining the impact of Budesonide-Surfactant vs. Surfactant alone on mortality in preterm neonates with NRDS, stratified by gestational age. Panel A reveals no statistically significant difference in mortality for neonates ≤27 gestational weeks (OR: 0.814, 95% CI: 0.589–1.125, *p* = 0.211), while Panel B demonstrates a significant reduction in mortality for neonates ≥27 gestational weeks receiving Budesonide-Surfactant (OR: 0.579, 95% CI: 0.378–0.887, *p* = 0.012). This suggests a gestational age-dependent effect of Budesonide-Surfactant on mortality, with a clear benefit observed in the more mature preterm population.

##### Mortality rate

**Figure 3:**
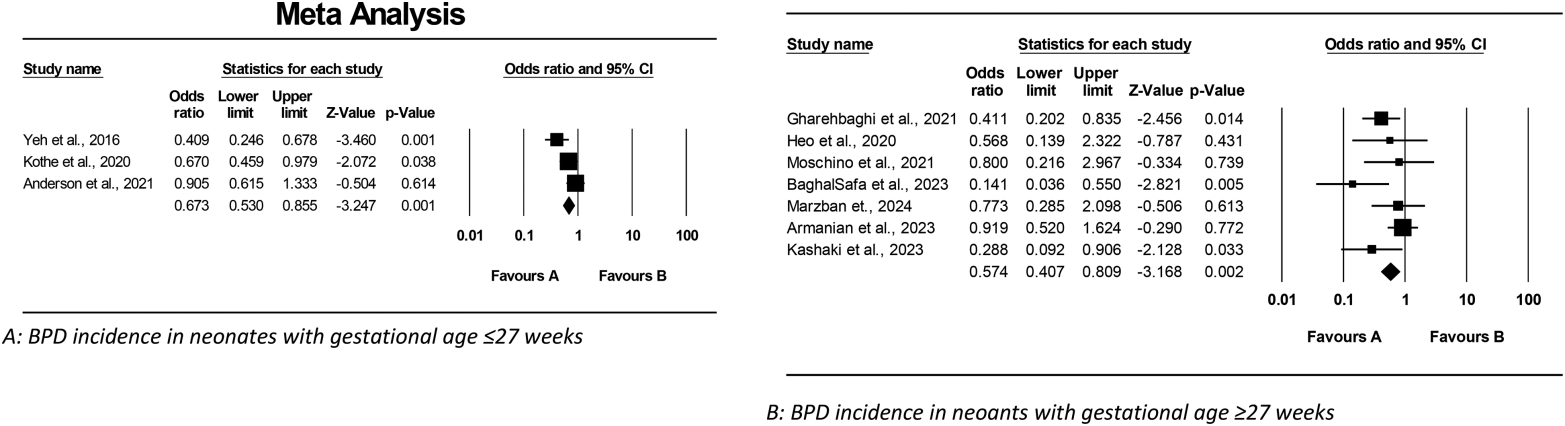
Meta analysis of the role of budesonide-surfactant mixture versus surfactant alone for prevention of BPD in preterm neonates with NRDS. **(A)** BPD incidence in neonates with gestational age ≤27 weeks. **(B)** BPD incidence in neoants with gestational age ≥27 weeks.

Figure 3 presents a meta-analysis assessing the impact of Budesonide-Surfactant vs. Surfactant alone on BPD incidence in preterm neonates with NRDS, stratified by gestational age; sub-analysis A demonstrates a significant reduction in BPD incidence among neonates ≤27 gestational weeks gestational age receiving Budesonide-Surfactant, while sub-analysis B similarly illustrates a significant reduction in BPD incidence in neonates ≥27 gestational weeks gestational age, thereby confirming a consistent protective effect of the Budesonide-Surfactant mixture against BPD across both gestational age groups.

### Results of meta-analysis of budesonide-surfactant effects on NRDS outcomes by gestational age

**Figure 4:**
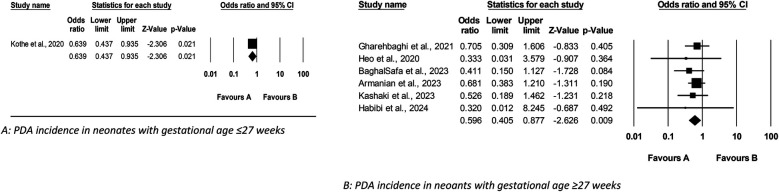
Meta analysis of the role of budesonide-surfactant mixture versus surfactant alone in incidence of PDA in preterm neonates with NRDS. **(A)** PDA incidence in neonates with gestational age ≤27 weeks. **(B)** PDA incidence in neoants with gestational age ≥27 weeks.

This meta-analysis examined the effect of Budesonide-Surfactant compared to Surfactant alone on primary and secondary outcomes in neonates with NRDS, stratified by gestational age (≥27 and ≤27 gestational weeks). In the ≥27 gestational weeks group (Tables 4, 5), Budesonide-Surfactant was associated with a statistically significant reduction in mortality (OR: 0.579, 95% CI: 0.378–0.887, *p* = 0.012), BPD (OR: 0.574, 95% CI: 0.407–0.809, *p* = 0.002), and PDA (OR: 0.596, 95% CI: 0.405–0.877, *p* = 0.009) (Figure 4). However, no significant differences were observed in ROP, NEC, IVH, or Sepsis. A slight, non-significant increase in Pneumothorax was observed. Heterogeneity was generally low to moderate across outcomes.

**Table 4:** Summary of primary and secondary complications in NRDS patients (gestational age ≥27).

Subgroup	Type of model	Heterogeneity		Odds ratio				Publication bias	
		*I*^2^ (%)	*P*_H_	OR	95% CI	*Z*_test_	*P*_OR_	*P*_Begg_	*P*_Egger_
Mortality	Fixed	0.000	0.852	0.579	0.378–0.887	−2.512	0.012	0.536	0.993
BPD	Fixed	37.186	0.145	0.574	0.407–0.809	−3.168	0.002	0.229	0.232
ROP	Fixed	45.678	0.118	1.029	0.664- 1.595	0.128	0.898	0.806	0.826
NEC	Fixed	0.000	0.575	0.669	0.402- 1.112	−1.551	0.121	0.806	0.078
IVH	Fixed	47.478	0.107	1.224	0.786–1.905	0.896	0.370	1.000	0.674
PDA	Fixed	0.000	0.933	0.596	0.405–0.877	−2.626	0.009	0.259	0.080
Sepsis	Fixed	79.405	0.028	0.903	0.461–1.877	−0.201	0.841	–	–
Pneumothorax	Fixed	19.111	0.293	1.719	0.977–3.024	1.881	0.060	0.220	0.033

**Table 5:** Summary of primary and secondary complications in NRDS patients (gestational age ≤27).

Subgroup	Type of model	Heterogeneity		Odds ratio				Publication bias	
		*I*^2^ (%)	*P*_H_	OR	95% CI	*Z*_test_	*P*_OR_	*P*_Begg_	*P*_Egger_
Mortality	Fixed	0.000	0.990	0.791	0.541–1.157	−1.209	0.227	0.601	–
BPD	Fixed	66.511	0.050	0.673	0.530–0.855	−3.247	0.001	1.000	0.342
ROP	Fixed	0.000	1.000	0.823	0.573–1.183	−1.051	0.293	–	–
NEC	Fixed	0.000	1.000	0.883	0.415–1.878	−0.324	0.746	–	–
IVH	Fixed	0.000	1.000	0.948	0.659–1.363	−0.289	0.772	–	–
PDA	Fixed	0.000	0.967	0.639	0.437–0.935	−2.306	0.021	–	–
Sepsis	Fixed	0.000	1.000	0.840	0.397–1.776	−0.458	0.647	–	–
Pneumothorax	Fixed	–	–	–	–	–	–	–	–

In the ≤27 gestational weeks group, a statistically significant reduction in BPD (OR: 0.673, 95% CI: 0.530–0.855, *p* = 0.001) and PDA (OR: 0.639, 95% CI: 0.437–0.935, *p* = 0.021) was also observed. No significant differences were found in mortality, ROP, NEC, IVH, or Sepsis. Pneumothorax data was unavailable for this group. Heterogeneity was moderate for BPD and low for the other outcomes. Overall, Budesonide-Surfactant demonstrated a consistent protective effect against BPD across both gestational age groups, with significant reductions observed. Mortality was significantly reduced in the ≥27 week group only.

## Discussion

BPD continues to pose a significant challenge in the management of preterm neonates, with potential long-term repercussions for respiratory health (27). Conventional approaches for treating NRDS, particularly traditional mechanical ventilation, can inadvertently inflict damage on the fragile lungs of premature infants (28). An emerging and promising strategy involves the combined use of budesonide and surfactant, which has demonstrated potential in mitigating the risk of BPD in this vulnerable population (28). Numerous studies have assessed the efficacy of budesonide-surfactant in preventing BPD among preterm infants, prompting our meta-analysis to evaluate the existing literature on this combined therapy (17).

This comprehensive study, combining a large-scale observational analysis with a rigorous meta-analysis, provides compelling evidence for the beneficial effects of Budesonide-Surfactant therapy in neonates with NRDS, stratified by gestational age. Notably, the consistent and significant reduction in BPD across both ≥27 and ≤27 weeks gestational age groups highlights the potential of this combined therapy to mitigate a major morbidity associated with prematurity. Furthermore, the observational data revealed significant reductions in several other morbidities, including NEC, PDA, ROP, and Sepsis, particularly in the ≥27 gestational weeks group, which were partially corroborated by the meta-analysis. The meta-analysis, focusing on mortality, BPD, and PDA, confirmed the significant protective effect of Budesonide-Surfactant, particularly in reducing BPD and PDA in both gestational age categories. While the observational analysis suggested broader benefits, the meta-analysis, with its inherent limitations and focus on select outcomes, primarily reinforced the robust findings regarding BPD and PDA. Of particular note, the significant reduction in mortality in the ≥27 gestational weeks group, as supported by both the observational and meta-analytic data, underscores the potential for improved survival in this population. However, in premature infants ≤27 weeks gestational age, no significant difference in mortality rates was observed. The observed increase in pneumothorax in the ≥27 gestational weeks group receiving Budesonide-Surfactant in the observational study warrants careful consideration and further investigation. Though the meta-analysis did not reveal a significant increase, the observational finding necessitates close monitoring of respiratory complications. The absence of pneumothorax data in the ≤27 gestational weeks group necessitates future research to assess this potential risk in the extremely preterm population.

This contrasts with a 2020 meta-analysis by He et al., which reported significant reductions in IVH, sepsis, and ROP complications while finding no significant difference in PDA incidence (12). The discrepancies may arise from variations in the studies included in each meta-analysis; while He et al. reviewed six studies, our analysis covered a broader range of literature. Similarly, a systematic review by Moraes LHA et al. included six studies, five of which were part of our analysis, with mixed findings regarding BPD or mortality reductions. Additionally, a limited analysis by Venkataraman et al. in 2016 involving two clinical trials indicated a 43% reduced risk of BPD with the budesonide-surfactant combination, though mortality outcomes were similar between groups (11). Overall, our research presents promising evidence that budesonide-surfactant therapy significantly reduces BPD incidence compared to standard care alone, aligning with findings by Gharehbaghi et al. on the benefits of budesonide as a rescue therapy for NRDS (27)..

The safety profile of budesonide-surfactant therapy is crucial. Most studies showed fewer side effects in the combination group, indicating it may be a safe alternative for preventing BPD in preterm neonates. Our findings highlight the benefits of this combination as an effective strategy for reducing BPD in high-risk preterm infants. Our meta-analysis of 11 clinical trials and observational studies revealed significant reductions in both BPD and mortality rates for those receiving this therapy compared to standard care. Although secondary outcomes like IVH, ROP, NEC, and sepsis did not show statistical significance, the trends are promising. The notable decrease in PDA occurrence further supports the benefits of this treatment approach. Importantly, our analysis indicates a favorable safety profile for budesonide-surfactant, with no new complications arising, suggesting it could be a valuable option for clinicians treating vulnerable preterm infants. Despite these positive findings aligning with existing literature, the variations in study outcomes emphasize the need for further research. Additional studies should clarify how budesonide enhances surfactant therapy and standardize treatment protocols. As we advance in BPD management, the budesonide-surfactant combination emerges as a significant intervention potentially leading to better respiratory outcomes and long-term health for this high-risk population. Future clinical trials should focus on the effectiveness and long-term implications of this regimen, including the optimal dosing, timing, and duration of therapy.

## Clinical implications and future directions

The findings of this meta-analysis emphasize the clinical implications of using budesonide-surfactant therapy as a promising intervention for reducing the incidence of BPD in preterm neonates, suggesting that it could become a standard treatment option in NICUs. Its favorable safety profile, combined with a significant reduction in BPD and mortality rates, highlights the potential for improving respiratory outcomes in this high-risk population. Future directions should focus on conducting larger, multicenter clinical trials to establish standardized treatment protocols, including optimal dosing, timing, and duration of the budesonide-surfactant regimen. Additionally, further research is necessary to investigate the mechanism by which budesonide enhances surfactant therapy and to clarify its effects on secondary complications such PDA and cardiac issues. This knowledge will contribute to optimizing therapeutic strategies and improving long-term health outcomes for vulnerable preterm infants.

## Limitations

This study has several limitations that must be acknowledged. Firstly, it focused on infants with NRDS and assessed the effects of the budesonide-surfactant combination solely in the context of preventing BPD and mortality, which may limit the generalizability of the findings. The included studies often had small sample sizes, and many did not report data blinding, raising concerns about potential selection bias. There is currently no standardized guideline for the dosage and administration frequency of budesonide and surfactant, which complicates the interpretation of results and may impact outcomes. Additionally, the side effects associated with varying doses of the combination therapy have not been thoroughly investigated, nor has the minimum effective dose for BPD prevention been established. Lastly, the potential positive effects of this treatment on cardiac issues remain less explored, indicating a need for further research in these areas to strengthen the conclusions drawn from this analysis.

## Conclusion

In conclusion, this meta-analysis indicates that combining budesonide with surfactant therapy significantly reduces the incidence of BPD and mortality rates in preterm infants with neonatal respiratory distress syndrome. The evidence shows favorable outcomes, including a marked decrease in PDA incidence and a generally good safety profile. While many secondary outcomes lacked statistically significant differences, there were positive trends in reducing complications like retinopathy of prematurity and sepsis. However, limitations such as small sample sizes, varied study designs, and the absence of standardized treatment protocols call for further research. Future studies should explore how budesonide enhances surfactant therapy and define optimal treatment parameters to improve outcomes for this vulnerable group. Overall, this combination therapy represents a notable advancement in managing BPD, potentially enhancing respiratory health and long-term outcomes for preterm neonates.

## Data Availability

The original contributions presented in the study are included in the article/Supplementary Material, further inquiries can be directed to the corresponding author.
